# Kinetics of Corrosion Inhibition of Aluminum in Acidic Media by Water-Soluble Natural Polymeric Pectates as Anionic Polyelectrolyte Inhibitors

**DOI:** 10.3390/ma6062436

**Published:** 2013-06-17

**Authors:** Refat M. Hassan, Ishaq A. Zaafarany

**Affiliations:** 1 Chemistry Department, Faculty of Science, Assiut University, Assiut 71516, Egypt; 2 Chemistry Department, Faculty of Applied Sciences, Umm Al-Qura University, Makkah Al-Mukarramah 13401, Saudi Arabia Kingdom; E-Mail: iazaafarany@uqu.edu.sa

**Keywords:** corrosion, inhibitors, aluminum, pectates, kinetics, mechanisms

## Abstract

Corrosion inhibition of aluminum (Al) in hydrochloric acid by anionic polyeletrolyte pectates (PEC) as a water-soluble natural polymer polysaccharide has been studied using both gasometric and weight loss techniques. The results drawn from these two techniques are comparable and exhibit negligible differences. The inhibition efficiency was found to increase with increasing inhibitor concentration and decrease with increasing temperature. The inhibition action of PEC on Al metal surface was found to obey the Freundlich isotherm. Factors such as the concentration and geometrical structure of the inhibitor, concentration of the corrosive medium, and temperature affecting the corrosion rates were examined. The kinetic parameters were evaluated and a suitable corrosion mechanism consistent with the kinetic results is discussed in the paper.

## 1. Introduction

Aluminum (Al) and its alloys are low cost and remarkable materials in industrial technology because of their light weight, high thermal and electrical conductivity as well as high resistance to corrosion in a wide variety of corrosive environments.

Generally, the corrosion resistance of metals, such as Al and steel, in corrosive environments, may be attributed to the formation of a protective tightly adhered invisible oxide film on the metal surface. The film reduces or prevents the corrosion of such metals. This film is generally stable in the solutions of pH ranges of about 4.5 to 8.5 [1]. However, due to the solubility of the film in strong acidic or alkaline solutions, the metal shows high rate of corrosion and dissolution in these conditions. Therefore, inhibitors are used to control both metal corrosion and acid consumption [2].

Although synthetic polymers [3,4,5,6], organic [7,8,9,10,11,12] and inorganic compounds [13,14] were applied as inhibitors to reduce the dissolution of Al in alkaline media, little attention has been focused on application of natural polymers as dissolution inhibitors in alkaline medium [15]. Hassan and co-workers investigated the corrosion inhibition of Al in NaOH by water-soluble alginates and pectates [16] as natural polymers carrying secondary alcoholic groups. Sulfated carrageenans [17,18,19,20] and carboxymethyl cellulose [21] as natural polymeric compounds, and polyacrylic acid [22] as a synthetic polymer containing alcoholic groups, have been successfully applied as corrosion inhibitors of steel in acidic media. However, the corrosion mechanisms, as well as the role of structure geometry and nature of the inhibitor on the corrosion processes, are still not completely understood.

In view of the above arguments and our interest in physicochemical properties of macromolecules, in particularly the natural polymeric compounds [23,24,25,26,27,28,29,30,31,32,33,34,35,36,37,38,39,40,41,42,43,44,45,46], the present work was undertaken to shed more light on the role of the nature of medium and the structural geometry of the inhibitor on the corrosion process. The study also aimed to elucidate a suitable mechanism for corrosion of Al in acidic medium and discuss the results in comparison with previously reported studies on use of the natural polymer pectate polysaccharide as inhibitor for corrosion of Al in alkaline solutions [16].

## 2. Results

### 2.1. Evolved-Hydrogen (and Weight Loss)—Time Curves

Corrosion inhibition performance of organic compounds can be evaluated using electrochemical and chemical techniques [6]. For the chemical methods, weight loss measurements are ideally suited for long term immersion test, whereas the gasometric technique is more suitable for short term immersion tests. The volume of evolved hydrogen (or weight loss of Al metal) as a function of time is defined as the rate of dissolution of Al in hydrochloric acid and can be expressed as Equations (1) and (2), respectively:

(1) $$R_{c}=\frac{V_{H_{2}}}{St}$$

(2) $$R_{c}=\frac{∆W}{St}$$

where, *R_c_* is the rate of corrosion, *S* is the surface area of Al metal (cm^2^), *t* is the time (min), $V_{H_{2}}$ is the volume of evolved hydrogen (mL) and ∆*W* is the loss in mass (mg) of Al metal in the corrosive medium.

### 2.2. Dependence of Corrosion Rate on [PEC]

**Figure 1:**
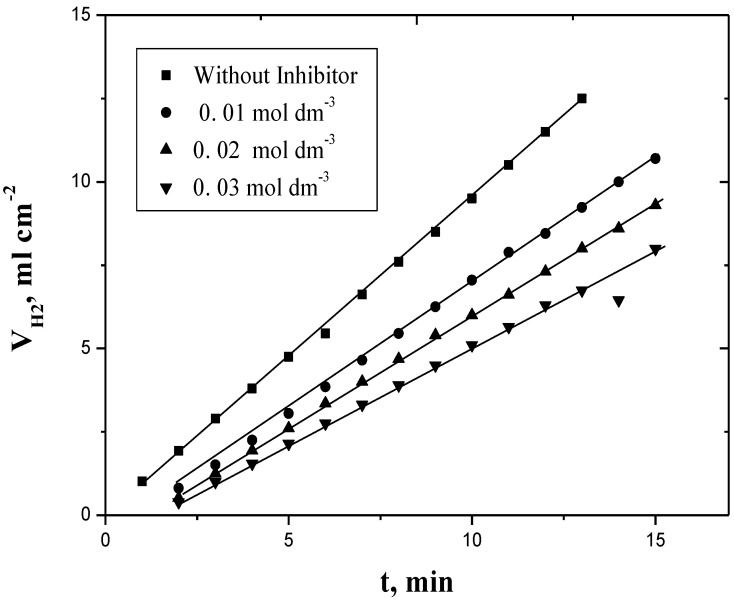
Plots of hydrogen evolved *vs.* time in the absence and presence of inhibitor for the corrosion of aluminum in HCl. [H^+^] = 4.0 mol dm^−3^ and I = 4.0 mol dm^−3^ at 30 ° C.

**Figure 2:**
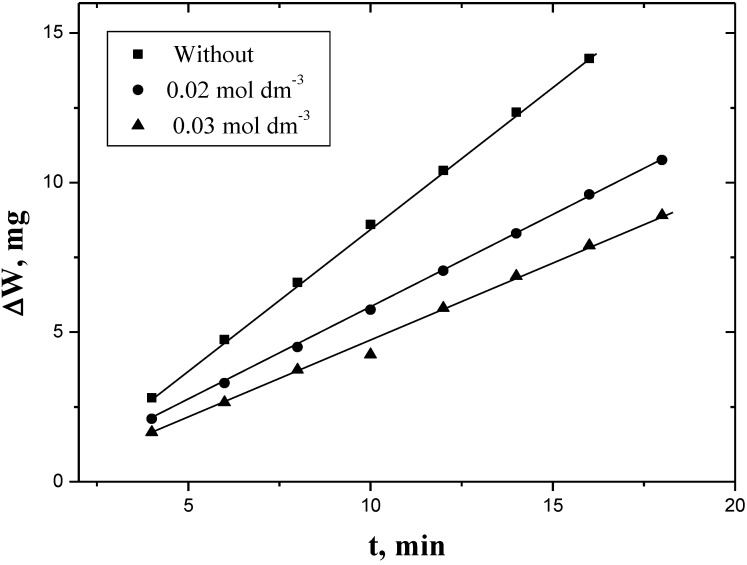
Plots of weight-loss *vs.* time in the absence and presence of inhibitor for the corrosion of aluminum in HCl. [H^+^] = 4.0 mol dm^−3^ and I = 4.0 mol dm^−3^ at 30 °C.

Plots of the evolved hydrogen or weight loss against time gave straight lines as shown in Figure 1 and Figure 2. The rate of corrosion (*R_c_*) was obtained from the slopes of such plots. The values of *R_c_* were calculated by using the method of least-squares and are summarized in Table 1. Increasing the concentration of PEC, keeping the concentrations of all other reagents constant resulted in decreasing the corrosion rate as shown in Table 1.

**Table 1:** Dependence of the corrosion rates (*R_c_*, mL cm^−3^ min^−1^ **)** on [PEC] for the corrosion of Al in HCl. [H^+^] = 4.0 and I = 4.0 mol dm^−3^ at various [PEC] and different temperatures.

[PEC]		Temperature		
% (w/v)	10^2^ mol dm^−3^	30 °C	35 °C	40 °C
0.0	0.0	0.95 (0.88) *	1.22	1.46
0.4	1.0	0.63 (0.59)	0.74	0.84
0.6	2.0	0.55 (0.50)	0.66	0.77
0.8	4.0	0.50	0.59	0.68
1.2	6.0	0.46	0.54	0.62

Experimental errors ±4%; * Values between parenthesis were evaluated from weight-loss method.

### 2.3. Dependence of Corrosion Rate on [H^+^]

In order to examine the influence of corrosion rates as a function of acid concentration, some experimental runs were performed at various initial concentrations of the acid and constants for all other reagents. The results are summarized in Table 2.

The percentage of inhibition efficiency (%IE) of PEC inhibitor was calculated by using the following equation

(3) $$\text{\%IE}=\frac{R_{c}^{o}-R_{c}^{\prime }}{R_{c}^{o}}\;\times 100$$

where, *R_c_^o^* and *R_c_′* are the corrosion rates of Al metal in the absence and presence of PEC inhibitor. The results are listed in Table 3.

**Table 2:** Dependence of the corrosion rate on [H^+^] for the corrosion of Al in HCl. [PEC] = 0.02 mol dm^−3^ (0.4%) at 40 °C.

[H^+^], mol dm^−3^	2.0	3.0	4.0	5.0
*R^o^_c_* (free), mL cm^−3^ min^−1^	0.39	0.77	1.46	1.98
*R′_c_* * (inh.), mL cm^−1^ min^−1^	0.13	0.46	0.84	1.20

Experimental errors ±4%; * [PEC] = 0.02 mol dm^−3^.

**Table 3:** Percentage inhibition efficiency (%IE) in the corrosion of Al in HCl. I = 4.0 mol dm^−3^ at various [PEC] and different temperatures.

[PEC]		30 °C [H^+^], mol dm^−3^		40 °C [H^+^], mol dm^−3^	
%(w/v)	mol dm^−3^	3.0	4.0	3.0	4.0
0.4	0.02	37.04	33.68	40.26	42.47
0.6	0.03	48.15	42.11	50.65	47.26
0.8	0.04	59.26	47.38	61.03	53.42
1.2	0.06	68.52	51.57	70.12	57.53

### 2.4. Dependence of Corrosion Rate on Temperature

In order to evaluate the kinetic parameters of the corrosion process, experimental measurements were performed at different temperatures keeping all other reagents concentration constant. The corrosion rates were found to increase with increasing the temperature as shown in Table 1.

## 3. Discussion

As shown in Table 1, addition of small amount of PEC solution to the HCl solution containing the test Al metal resulted in a remarkable decrease in the corrosion rate of Al metal. The corrosion rate was found to be a function of the concentration of the acid. This result indicates that at least one of the corrosion paths of dissolution of Al metal in HCl solution should involve the presence of hydrogen ions in the rate-determining step.

Moreover, the inhibition efficiency (%IE) increased with increasing the concentration of the added inhibitors in Table 3. The inhibition efficiency may be affected by many factors, such as the adsorption of the additives on Al metal surface, which depend on some physicochemical properties, e.g., the functional groups, steric factors and electronic and the geometrical configurations of the inhibitor [16,17,18].

### 3.1. Corrosion Mechanism

We propose a suitable mechanism of corrosion, in accordance with the above experimental observations. The corrosion of metal involves an electrochemical process [47,48,49] resulting from dissolution of Al metal in the acid. This process can be expressed by the anodic and cathodic processes, which are defined by Equations (4) and (5), respectively,

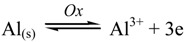
(4)

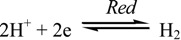
(5)


The overall electrochemical process can be written as follows:

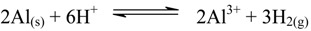
(6)


The cathodic reaction produces H_chemisorbed_ by picking up an electron that released in the anodic reaction (H^+^ + e = H_chemisorbed_ ) in Al corrosion in HCl. In such acidic solutions, the H_chemisorbed_ on the metal surface reacts by combining with other adsorbed H_chemisorbed_ to form H_2_ gas molecule, which bubbles from the metal surface. A very small amount of the uncombined H_chemisorbed_ will remain; however, this amount does not affect the whole process. Therefore, the rates of combination and absorption of H_chemisorbed_ are nearly the same for all inhibitor levels. This fact is confirmed by the identical results obtained for the corrosion rates calculated from the gasometric and weight loss techniques in Table 1.

It has already been reported [50] that an inhibitor can affect the corrosion rate of metals in a corrosive medium if the inhibitor is able to affect the kinetics of dissolution or alter the position of electrochemical behavior. This phenomenon takes place when a thin film of the inhibitor is formed on the metal surface either by the interaction or the adsorption processes. A thin film is usually formed by the adsorption of anionic inhibitors on the positive sites formed on Al surface as a result of liberation of electrons in the anodic process. The protective coating of the thin film will isolate the Al metal from the corrosive medium and, this prevents more Al atoms from leaving the metal surface to the corrosive medium thereby decreasing the rate of corrosion. Therefore, the anodic reaction may be considered as the rate-determining step in the corrosion process.

Khairou and El-Sayed [51] reported that the presence of functional hydroxyl groups within the inhibitor macromolecules could make bridges between the polymer and the metal surface and, as a result, the rate of corrosion decreases. Moreover, the presence of lone-pairs of electrons on the oxygen atoms of the hydroxyl groups of the inhibitor may enhance the interaction between the inhibitor and the positive sites formed on Al surface.

### 3.2. Adsorption Isotherm

In aqueous solution, the metal surface is always covered with adsorbed water molecules. Therefore, the adsorption of inhibitor molecules from an aqueous solution is a quasi substituted process [52] and the inhibitors that have the ability to adsorb strongly on the metal surface will hinder the dissolution reaction of such metal in the corrosive medium. Here, the degree of surface coverage (*θ*) is considered as the determining factor that plays the main role in inhibition efficiency [53]. The value of (*θ*) can be evaluated from the following relationship:

*θ= 1 –* (*R′_c_*)_inh_/(*R^o^_c_* )_free_ (7)

where (*R′_c_*)_inh_ and (*R^o^_c_*)_free_ are the corrosion rates in the presence and absence of inhibitor. It was observed that θ values decreased with increase in temperature as a result of increased e adsorption of inhibitor molecules. On the other hand, the *θ* values increased with increase in inhibitor concentration as a result of decrease in the corrosion rates *R*_cinh_ (numerator in Equation (7)).

Theoretically, the adsorption process can be regarded as a single substitution of (X) molecule of the water molecules adsorbed on the metal surface by the following reaction:

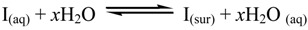
(8)
where *x* is the size ratio and equals the number of adsorbed water molecules replaced by a single inhibitor molecule. The extent of adsorption depends on many factors, such as the nature of metal, conditions of metal surface, the chemical structure of the inhibitor and the nature of its functional groups, pH and type of corrosion medium and temperature [17,18,19,54].

The adsorption also provides some information about the interaction among the inhibitor molecules themselves as well as their interaction with the metal surface. Actually, the adsorbed molecules may cause some difficulty for the surface to adsorb further molecules from neighboring sites and hence, a multilayer-adsorption may take place. The net result is the formation of various surface sites with varying the degrees of activation. For this reason, a number of mathematical adsorption expressions have been developed to fit the degree of surface coverage through adsorption isotherms in order to provide some knowledge on the nature of interaction of the adsorbed molecules.

**Figure 3:**
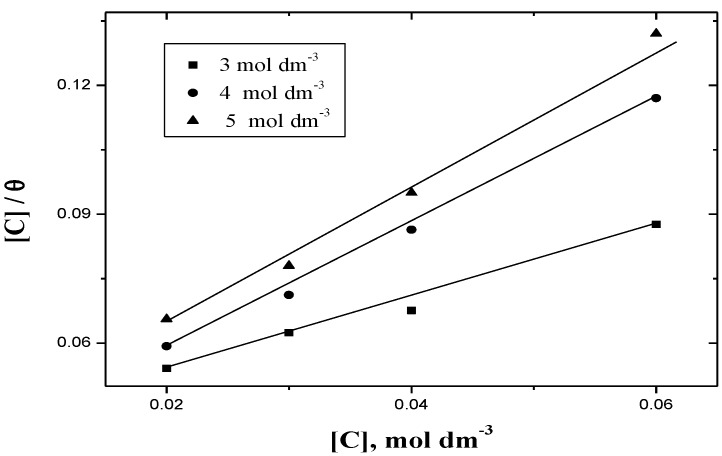
Plots of *[C]/θ* *versus* *[C]* of Langumir’s adsorption isotherm for the corrosion of aluminum in HCl. I = 4.0 mol dm^−3^ and various acid concentrations at 30 ° C.

Langmuir isotherm suggests that each site holds one adsorbed species [14,53,54,55,56,57] and can be represented by Equation (9).

(9) $$\frac{C}{\theta }=\frac{1}{K_{ads}}+C$$

where *C* is the concentration of inhibitor and *K_ads_* is the equilibrium constant of adsorption process. Equation (9) required that a plot of *C/θ* against *C* should be linear with a positive intercept on *C/θ* axes and of unity slope. The experimental data were found to satisfy this requirement with good correlation coefficients, but the slopes were deviated from the unity values as shown in Figure 3. The deviation from unity slope shows that Langmuir isotherm may not be strictly applied in this case. The experimental results of the present study were tested by fitting to Freundlich adsorption isotherm [19,58] which is defined by the following relationship:

log*θ =* log*K_ads_ + n*log*[C]* (0 < *n* < 1) (10)

**Figure 4:**
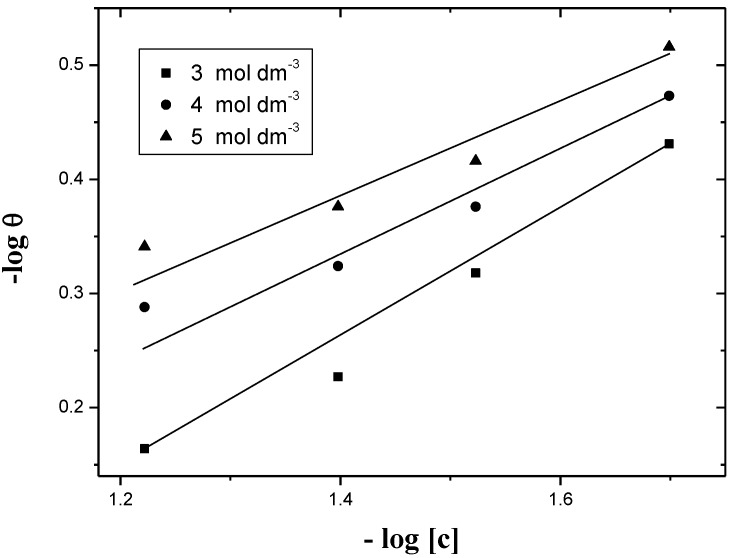
Plots of log*θ vs.* log*[C]* of Freundlich adsorption isotherm for the corrosion of aluminum in HCl. I = 4.0 mol dm^−3^ and various acid concentrations and at 30 °C.

According to Equation (10), when log*θ* is plotted against log*[C]*, it should give straight lines with intercepts on log*θ* axis as was experimentally observed in Figure 4. The values of *n* and *K_ads_* can be evaluated from the slopes and intercepts of such plots, respectively. These values were calculated by the method of least-squares and are summarized in Table 5.

The standard adsorption free energy ($∆{\text{G}}^{0}$) can be calculated from the well-known relationship that relates the adsorption equilibrium constant to the adsorption free-energy [54,55,59] as follows,

(11) $$\log K_{ads}=-\log C_{H_{2}O}-{\left(\Delta {\text{G}}^{\text{0}}\right)}_{ads}/2.303RT$$

where ${\text{C}}_{\;H_{2}O}$ is the molar concentration of water (55.5), R is the molar gas constant and T is the absolute temperature. Thermodynamically, ∆G^0^_ads_ is related to the enthalpy (∆H^0^_ads_) and entropy (∆S^0^_ads_) of the adsorption process by the famous Gibbs- Helmholtz equation

∆*G*^0^*_ads_* = ∆H^0^*ads−T*∆S^0^*_ads_* (12)

From Equations (11) and (12), the following relationship may be deduced [56,57],

(13) $$\text{log}K_{ads}=(-\text{log}C_{{\text{H}}_{2}\text{O}}+\frac{∆{\text{S}}_{\text{ads}}^{0}}{2.303\text{R}})-\frac{∆{\text{H}}_{\text{ads}}^{0}}{2.303\text{RT}}$$

Equation (13) required that plots of log*K_ads_* *vs.* *1/T* to be linear as was observed experimentally. The values of ∆H^0^_ads_ and ∆S^0^_ads_ can be evaluated from the slopes and intercepts of such plots. These values were calculated by using the least-squares method and are summarized in Table 4. The observed negative values of ∆G^0^_ads_ indicated that the adsorption process of PEC inhibitor on Al surface is a spontaneous process.

**Table 4:** Thermodynamic parameters for the corrosion of Al in HCl. I = 4.0 mol dm^−3^, [H^+^] = 4.0 mol dm^−3^ at 30 °C.

Parameter	Slope (n) 0.57	Slope (n) 0.39
10^2^ K, dm^3^ mol^−1^	28	62
−ΔG^0^, kJ mol^−1^	6.91	2.81
−ΔH^0^, kJ mol^−1^	15.28	16.05
−ΔS^0^, J mol^−1^ K^−1^	73.30	82.40

**Figure 5:**
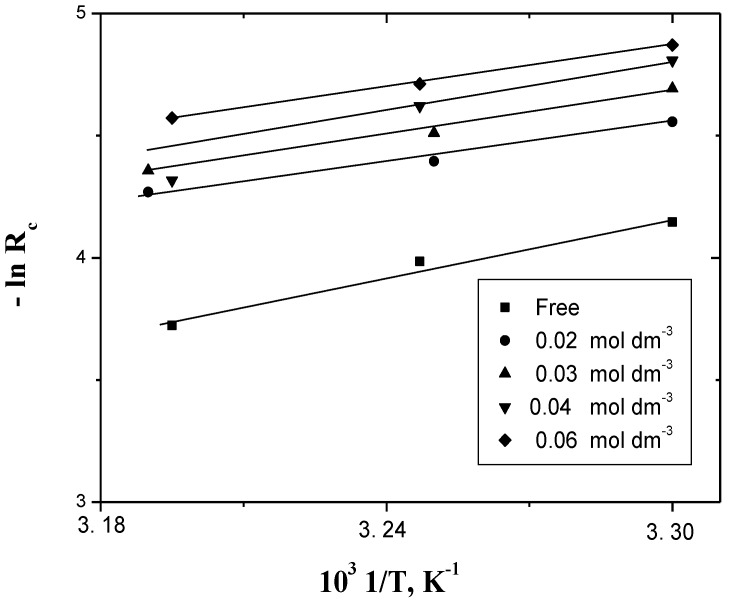
Arrhenius plots of the temperature-dependence for the corrosion rate in the corrosion of aluminum in HCl. [H^+^] = 4.0 and I = 4.0 mol dm^−3^ at various [PEC] and different temperatures.

**Figure 6:**
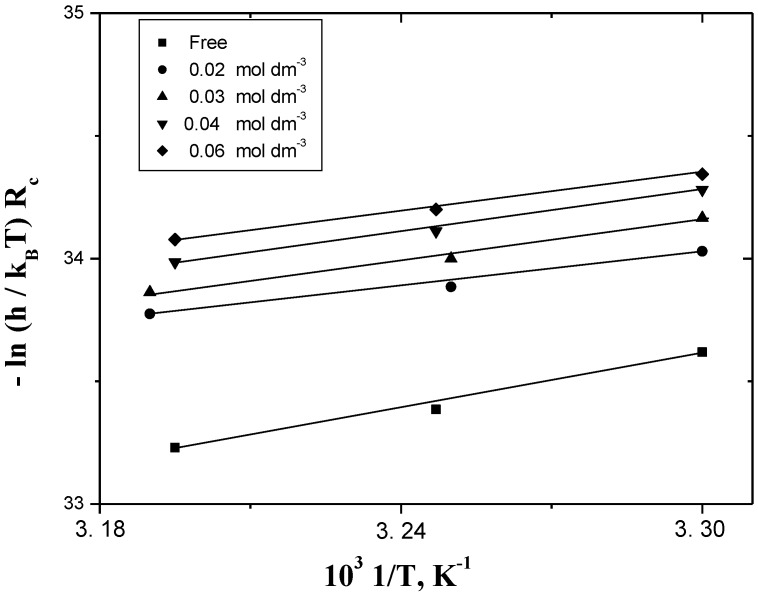
Eyring plots of the temperature-dependence for the corrosion rate in the corrosion of Al in HCl. [H^+^] = 4.0 mol dm^−3^ and I = 4.0 mol dm^−3^.

The activation parameters of the corrosion inhibition were calculated from the dependence of the corrosion rate on temperature. This dependence was found to fit the Arrhenius and Eyring relationships [60] defined by Equations (14) and (15), respectively:

(14) $$\ln R_{c}=\ln A-\frac{E^{\neq }}{RT}$$

where, *A* is the frequency factor, *E^≠^* is the apparent activation energy, *R* is the gas constant and *T* is the absolute temperature, and

(15) $$-\ln\frac{Rh}{NT}R_{c}=\frac{\Delta H^{\neq }}{RT}-\frac{\Delta S^{\neq }}{R}$$

where *h* is the Planck’s constant, *N* is the Avogadro’s number, ∆*H^≠^* is the enthalpy of activation and ∆*S^≠^* is the entropy of activation. The kinetic results were found to fit the Arrhenius and Eyring equations, where plots of *1/T vs. −*ln*R_c_* or *1/T vs. −*ln*(hR_c_/k_B_T)* ((*k_B_* is Boltzman constant and equals the term *R/N*) resulted in good straight lines. The activation parameters ∆*H^≠^* and ∆*S^≠^* can be evaluated from the slopes and intercepts of the straight line, respectively, as shown in Figure 5 and Figure 6.

These values were calculated by using the least-squares method and are summarized, along with the reported corrosion inhibition of Al in alkaline medium, in Table 5. The positive values of ∆H^#^ reflect the endothermic process of adsorption of the inhibitors on Al surface. The negative values of ∆S^#^ may reflect the association mechanism of corrosion, *i.e.*, the decrease in disorder takes place on going from reactants to the activated states [16,17].

It has also been noticed that the addition of small amount of the inhibitor to the test solution alters the magnitude of ∆*S^≠^* (in absence of inhibitors) to a less negative value, *i.e.*, decreases the corrosion rates. This result may be considered as indirect evidence to support the cited proposed mechanism.

**Table 5:** Activation parameters for the corrosion of Al in HCl in the presence and absence of added inhibitors.

Parameter [PEC]		Δ*H^≠^* kJ mol^−1^	Δ*S^≠^* J mol^−1^K^−1^	Δ*G^≠^* kJ mol^−1^	*E_a_^≠^* kJ mol^−1^	A mol^−1^ s^−1^	*R_c_* * mL cm^−2^ min^−1^	%IE *
%(w/v)	mol dm^−3^							
0	0	30.90	−177.44	83.78	33.51	9.51 × 10^3^	0.95	–
0.4	0.02	20.20	−216.22	84.63	22.73	0.87 × 10^2^	0.70	22.22
0.6	0.03	24.8	−204.57	85.04	26.61	3.54 × 10^2^	0.63	30.00
0.8	0.04	23.37	−207. 84	85.31	24.08	1.19 × 10^2^	0.50	44.44
1.2	0.06	21.07	−215.99	85.43	23.66	0.91 × 10^2^	0.46	48.89
**Activation parameters in alkaline solutions [16]**								
0	0	58.85	−61.97	77.31	61.15	1.11x10^11^	0.63	–
0.4	0.02	88.35	+ 3.69	87.31	91.06	6.83x10^13^	0.32	46.67
0.8	0.04	92.63	+ 11.13	89.31	95.15	6.46x10^13^	0.16	72.67
1.2	0.06	88.82	−4.77	90.42	91.78	1.16x10^13^	0.11	81.67

Experimental Errors ±4%; * [Medium] = 4.0 mol dm^−3^ at 30 °C.

Furthermore, the values of E^≠^ for the inhibited solutions are higher than that of the uninhibited ones, indicating the inhibitive acts by decreasing the energy barrier for the corrosion process. This emphasizes the electrostatic character of the adsorbed inhibitor on Al surface. Again, the observed decrease in the apparent activation energy, *E^≠^*, at higher inhibitor efficiency may arise from the shift of the net corrosion reaction from that on the uncovered surface to one involving the adsorbedsites [56,57,61,62,63,64].

**Scheme 1:**
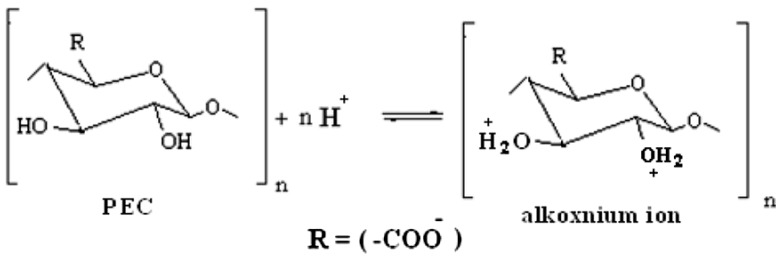
Protonation of PEC in acidic medium.

The inhibition efficiency, rate of corrosion and kinetic parameters for corrosion inhibition of Al in acidic and alkaline media by anionic-polyelectrolytes such as pectates, a natural polymer are compared in Table 5. It is evident from the results that the influence of pectates as an inhibitor for the corrosion of Al in alkaline medium is more effective than that in acidic medium. This behavior can be interpreted by the fact that sodium pectate, a water-soluble natural polymer with linear block copolymer structures carrying secondary alcoholic functional groups has a high tendency for protonation in acidic solutions to give its corresponding positive alkoxnium ions [45,46] as shown in the following (Scheme 1)

These positive alkoxnium species may hinder the formation of positive sites on Al surface as a result of the anodic process. This in turn may affect the adsorption of inhibitor molecules.

## 4. Experimental Section

### 4.1. Materials

All the materials used were of analytical grade. Bi-distilled water was used in all preparations. Sodium pectate (Fluka) was used without further purification. Purity of Al metal used was 99.98% (Ventron Corp., Osaka, Japan).

### 4.2. Preparation of Pectate Sols

Pectate sols (PEC) were prepared as described elsewhere [45,46]. This process was performed by stepwise addition of the powder reagent to bi-distilled water whilst vigorously stirring the solutions to avoid the formation of lumpy precipitates, which swell with difficulty.

### 4.3. Hydrogen Evolution Measurements

This technique provides a rapid and reliable means of assessing the inhibitive performance of Al in acidic solutions at short time immersions. The rates of corrosion were determined volumetrically by measuring the evolved hydrogen produced from dissolution of Al in HCl as a function of time. Rectangular specimens of Al metal of 3 cm long and 1.9 cm in diameter were used without further polishing to ensure reproducible surface. They were washed with carbon tetrachloride, absolute ethyl alcohol and then dried in acetone and stored in moisture-free desiccators prior to their use in corrosion testing. The specimen were suspended by means of glass hook in the tested solutions of HCl in conical flask fitted with graded side-arm burette filled with bi-distilled water as described elsewhere [21,22]. The conical flasks were thermostated in a controlled water-bath at the desired temperature within ±0.1 °C. When the HCl solution attained the temperature of the thermostat, the Al specimens were immersed into the acid solution. The course of reaction was followed gasometrically by recording the volume of evolved hydrogen as a function of time. The volume of active hydrogen was evaluated in accordance to the dimensions of Al specimen plates used.

Kinetic measurements were performed using the classical weight-loss method [47,48] in order to check the reproducibility of the gasometric data. The results obtained were found to be in a good agreement with each other within the experimental errors (±5%). This fact may indicate the reproducibility of the results obtained by the gasometric technique.

All the experiments were repeated using different concentrations of HCl and inhibitor at various temperatures. The results reported here are the averages of at least five experimental runs. The corrosion medium was not stirred during the test. The ionic strength was maintained constant at 4.0 mol dm^−3^ by adding NaClO_4_ as an inert electrolyte.

## 5. Conclusions

Anionic polyelectrolyte pectates as a natural polymer may be considered as a safe and effective inhibitor for decreasing the corrosion of Al in acidic medium. The geometrical configuration and functional groups within the inhibitor molecule are the two main important factors to influence the inhibition efficiency. We also demonstrated higher inhibition efficiency of pectates for Al dissolution in alkaline solution compared with that in an acidic medium.
